# Are Nanobiosensors an Improved Solution for Diagnosis of *Leishmania*?

**DOI:** 10.3390/pharmaceutics13040491

**Published:** 2021-04-03

**Authors:** Sona Jain, Wanessa Santana, Silvio S. Dolabella, André L. S. Santos, Eliana B. Souto, Patrícia Severino

**Affiliations:** 1 Postgraduate Program in Industrial Biotechnology, Universidade Tiradentes, Aracaju 49032-490, Brazil; wanessa-santanam@hotmail.com (W.S.); patricia_severino@itp.org.br (P.S.); 2 Department of Morphology, Federal University of Sergipe, São Cristóvão 49100-000, Brazil; dolabella@ufs.br; 3 Paulo de Góes Microbiology Institute, Departament of General Microbiology, Federal University of Rio de Janeiro, Rio de Janeiro 21941-901, Brazil; andre@micro.ufrj.br; 4 CEB—Centre of Biological Engineering, University of Minho, Campus de Gualtar, 4710-057 Braga, Portugal; 5 Department of Pharmaceutical Technology, Faculty of Pharmacy, University of Coimbra, Pólo das Ciências da Saúde, Azinhaga de Santa Comba, 3004-531 Coimbra, Portugal

**Keywords:** leishmaniasis, nanomaterials, immunosensors, genosensors, parasitic diseases

## Abstract

Leishmaniasis is one of the deadliest neglected tropical diseases affecting 12–15 million people worldwide, especially in middle- and low-income countries. Rapid and accurate diagnosis of the disease is important for its adequate management and treatment. Several techniques are available for the diagnosis of leishmaniasis. Among these, parasitological and immunological tests are most widely used. However, in most cases, the utilized diagnostic techniques are not good enough, showing cross-reactivity and reduced accuracy. In recent years, many new methods have been reported with potential for improved diagnosis. This review focuses on the diagnosis of *Leishmania* exploring the biosensors and nanotechnology-based options for their detection. New developments including the use of nanomaterials as fluorophores, fluorescence quenchers as reducing agents and as dendrimers for signal improvement and amplification, together with the use of aptamers to replace antibodies are described. Future research opportunities to overcome the current limitations on the available diagnostic approaches are also discussed.

## 1. Introduction

Leishmaniasis is one of the seven most important tropical diseases in the world. It is caused by the obligate intracellular parasitic protozoa belonging to the genus *Leishmania* and affects millions of people in the world. In 2018, leishmaniasis was reported in 98 countries and territories across four continents. With approximately 1–2 million new cases and 70,000 deaths every year, it is among the deadliest of the neglected tropical diseases (NTDs) [1,2].

**Figure 1:**
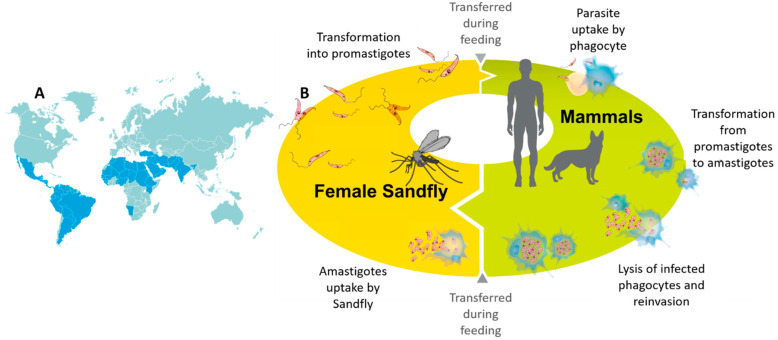
Geographical distribution of leishmaniasis in the world (**A**). Life cycle of *Leishmania* in sand fly and mammalian host (**B**). The two main evolutionary forms during their life cycle: the promastigote (in the invertebrate host) and the amastigote (present in the vertebrate host) are shown.

Leishmaniasis usually affects underdeveloped countries in Africa, Asia, and Latin America (Figure 1A), and is related to malnutrition, population migration, poor living conditions, fragile immune system, and lack of resources [3]. Recent surveys indicate that approximately 350 million people live in a vulnerable situation with the risk of contracting leishmaniasis. From a global perspective, the disease currently affects approximately 12–15 million people worldwide [4].

There are more than 20 *Leishmania* species distributed worldwide that are transmitted by over 90 phlebotomine sandfly species [5]. Leishmaniasis is usually transmitted by the bite of an infected female sandfly to mammalian host, such as rodents, marsupials, edentates, monkeys, and wild or domestic canines (Figure 1B). Humans are accidentally infected in endemic areas [6]. Distinct species of *Leishmania* can cause different clinical manifestations, and the disease can be grouped into three main clinical forms: cutaneous leishmaniasis (CL), mucocutaneous leishmaniasis (ML), and visceral leishmaniasis (VL), also known as kala-azar [3]. Each form varies in the degree of severity, where VL is the most severe form presenting the highest mortality.

CL is the most prevalent form caused by leishmanial species, such as *L. tropica*, *L. amazonensis*, *L. aethiopica*, and *L. major*, and it is generally painless and chronic, resulting in constant scar and critical stigma or disability [7]. ML is most frequently found in Americas and is caused by species such as *L. braziliensis* and *L. mexicana*. ML is characterized by the appearance of lesions that may partially or completely destroy the mucous membranes of the cavities of the nose, mouth, and throat, leading to disfigurement of the patient with consequent social exclusion [8]. As mentioned before, VL is the most severe form of the disease caused by *L. donovani* and *L. infantum*, and it is characterized by fever, weight loss, enlargement of liver and spleen, pancytopenia, and hypergammaglobulinemia and may be fatal if left untreated [9]. 

Early treatment as well as rapid and accurate diagnosis of the disease are important for its control [10,11]. Parasitological, serological, and molecular methods currently in use for diagnosis still have limitations that make the confirmation of the disease difficult [12]. The parasitological diagnosis, in addition to being invasive, shows low sensitivity. Serological tests work well in people who have a good immune response, but immuno-depressed patients, for example, do not respond well due to deficiencies in antibody production. Molecular tests, although of high precision, generally depend on equipment that is not always available [13]. The current diagnostic methods are thus often not sufficient, being presumptive, and diagnosis in most cases usually involves the identification of the *Leishmania* species mainly based on local epidemiology and clinical aspects [14]. This generates an urgent need to develop more sensitive, specific, and accessible tests, with easy application in the field, especially in poorly or modestly equipped laboratories. 

Recent years have seen a great advancement in the development of nanomaterials and bioanalytical chemistry. Many emerging methods have been described based on nanomaterials such as optical labels (Hu et al. 2017), use of new signal transduction mechanisms [15], and use of aptamers over antibodies [16,17]. In this review, we discuss the state-of-the-art on the use of nanomaterials for the detection of leishmanial parasites and the role of surface-tailored aptamers for the improved diagnosis of the disease.

## 2. Current Diagnostic Methods and Limitations

The control of leishmaniasis requires a combined set of intervention strategies, among which early diagnosis and treatment are important aspects. Current diagnosis is mostly based on the use of immunological and parasitological tests combined with clinical symptoms. However, in the case of CL and ML, serological tests have limited value. 

### 2.1. Parasitological Tests 

Parasitological tests are still considered the gold standard for the diagnosis of leishmaniasis. In this direct identification method, diagnosis is made microscopically by identifying amastigotes in affected tissues (CL/ML injury site) or in sample aspirated from the spleen, bone marrow, or lymph nodes in case of VL patients [10,11,14]. The inoculation of samples in experimental animals (xenodiagnoses), as well as the in vitro culture of *Leishmania* promastigotes in culture medium, are also used in the routine diagnosis of leishmaniasis. A combination of microscopy and culture methods enhances the diagnostic sensitivity by more than 85% [14,18]. However, all these methods are expensive and time-consuming, require skilled labor, and do not discriminate between *Leishmania* species [10,11].

### 2.2. Immunological Tests

The leishmania skin test or Montenegro test is used to measure the delayed-type hypersensitivity reaction to an intradermal injection of a suspension of killed *Leishmania* promastigotes [10,11]. It is a simple, sensitive, and specific method for CL; however, it does not allow the identification of species and does not differentiate past infections from present infections [10]. In addition, the test gives negative results during the disease period in VL [11]. 

Serological tests like indirect immunofluorescence assay (IFA), immunoenzymatic assay (ELISA), and the immunochromatographic test (IC) are also frequently utilized for the diagnosis of leishmaniasis. Other less employed serological tests include direct agglutination test (DAT), fast agglutination screening test (FAST), the complement fixation reaction (CFR), and western blot (WB). The sensitivity and specificity of these methods are directly related to the technique used and the manifestation of the disease [11,19]. Moreover, these antibody detection tests do not differentiate between recent and past infections, as the antibodies remain positive months after curing the patient [14]. The IFA test shows acceptable sensitivity (87–100%) and specificity (77–100%) [8,20,21]. However, the need for a fluorescence microscope limits the use of the IFA test to reference laboratories [14]. ELISA is the preferred laboratory test for serodiagnosis of VL. The technique is highly sensitive, but its specificity depends upon the antigens used. Variations in sensitivity (80–100%) and specificity (71–100%) are reported in ELISA tests using crude *Leishmania* spp. antigens [22,23,24]. The use of recombinant or purified antigens, such as membrane glycoproteins gp63, gp72, gp70, and rK39 specific to the Leishmania genus, improve the sensitivity and specificity of the technique. However, cross-reactions with diseases caused by other trypanosomatids can still occur [23]. In view of the need to achieve high precision, several new serological methodologies have been developed in recent years, and research using rapid immunochromatographic tests has been explored. Among these, the point of care (POC) tests using different antigens are popular, however they show varying sensitivity and specificity among different populations [14]. With respect to cytometry, although the technique has also shown high sensitivity and specificity, and presents advantages over other immunoassays, it is costly and requires specialized technicians to manipulate the equipment. Thus, its use in diagnostic routine is limited and is usually intended for large research centers [25,26,27].

### 2.3. Molecular Tests

Polymerase chain reaction (PCR) is a highly sensitive and important method used for the diagnosis of leishmaniasis, mainly in immunosuppressed patients. PCR can be performed using a wide variety of samples, such as blood, spleen, and lymph node [10,14]. One of its main advantages is the possibility of quantifying the parasitic load in the samples (qPCR), allowing the progression of the disease to be monitored and, consequently, the effectiveness of the anti-Leishmania therapy used [10,14]. Molecular techniques, although not used in the routine diagnosis, are highly sensitive. PCR has a reported specificity of 100% (for CL) with an improved sensitivity of 20 to 30% when compared with conventional parasitology diagnosis, and thus its inclusion as one of the routinely diagnostic methods could be highly beneficial. Real-time quantitative PCR (qPCR) has been shown to have high analytical sensitivity (0.0125 parasites per mL of blood) and excellent linearity [28]. PCR allows identification of the species of *Leishmania* that is causing the infection [10,29]. However, the lack of standardization in the protocols for the different reaction steps (obtaining the samples, extracting the DNA, and selecting the primers) makes it difficult to implement the technique on a large scale [11,30]. Furthermore, these tests remain limited to reference hospitals and research centers because of high costs and the requirement for special equipment and skilled personnel. Efforts are needed to make PCR more economical and easier to use, especially in endemic areas [11,14]. Despite the great progress, there is still no standard test for the diagnosis of leishmaniasis. An effective diagnostic method, needs to be sensitive, specific, fast, accessible, and easy to use, among other factors. Together, these factors will result in early efficient disease identification. 

## 3. Emerging Nanomaterial-Based Detection Technologies

The limitations encountered in the methods currently in use for the diagnosis of *Leishmania* demand development of new, efficient, rapid, and innovative POC approaches. Over the years, biosensors and nano-based technologies have proven to be promising options to meet this ever-increasing demand. Biosensors consist of a biomolecule (antigen, antibody, oligonucleotide, or enzyme) as recognition element coupled to a signal transducer. Binding of the recognition element to the target generates a signal by the transducer [31]. Based on the recognition element, biosensors can be categorized as immunosensors, genosensors, and aptamer-based sensors [32]. Immunosensors use antibodies as the recognition element. The transducer in this case converts the antibody–antigen interaction into a measurable physical signal. Genosensors, on the other hand, are DNA biosensors involving a hybridization reaction between two complementary oligonucleotides [33]. In this case, the use of nucleic acids eliminates the need for expensive antibodies, resulting in higher stability and discarding the need for special storage. Aptamer-based sensors make use of aptamers (single-stranded DNA oligonucleotides that can specifically bind target analytes) also known as chemical antibodies [34].

Nanomaterials are known for their excellent optical, catalytic, electrical, and magnetic properties [35,36,37,38,39]. Many published reports describe the use of these special physical and chemical characteristics of nanomaterials for the development of biosensors and other nano-based tools for the detection of *Leishmania* which will be described in this section (Table 1).

### 3.1. Nanomaterials as Fluorescence Quenchers

Various types of nanostructures such carbon nanotubes; graphene oxide [54,55]; Au [56]; Ag [57,58] and ceria [59] nanoparticles; quantum dots [60]; metal–organic structures [61,62]; transition metal nanosheets [63,64]; and conductive polymers—such as poly(3,4-ethylenedioxythiophene) (PEDOT) nanoparticles [65], polypyrrole (PPY) nanospheres [66], and polyaniline (PANI) nanofibers, have been reported as effective fluorescence quenchers. Immobilizing these nanostructures on flexible substrates and their further integration in portable devices have been explored in many different applications. Functionalized nanostructures (with the probe of interest) have been used as fluorescence test strips, reducing the time required to carry out a diagnostic assay.

**Figure 2:**
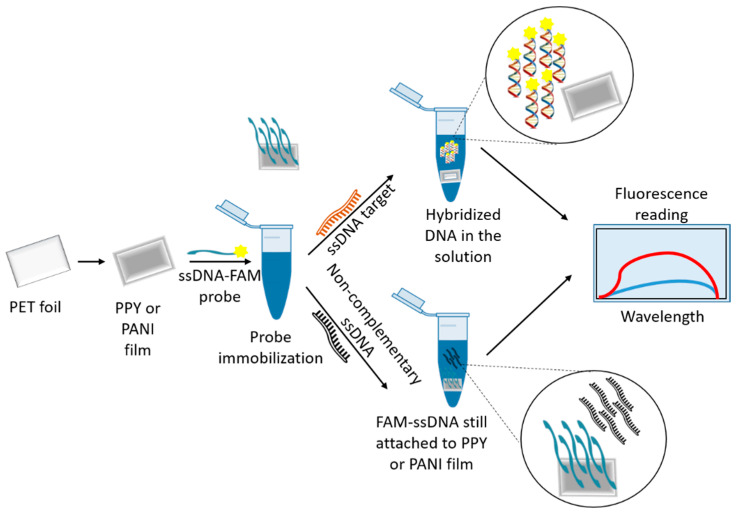
Nanostructured films as quenchers for fluorescent detection. Mechanism of fluorescence detection (of nucleic acids) using nanostructured ICP/PET films as sensing platform is demonstrated.

Pedro et al. (2019) [67] described the use of nanomaterials as nanoquenchers for fluorescent DNA assays. The nanostructure developed interacted with the fluorophore of a labeled DNA probe (*L. infantum* specific) through electron transfer processes which resulted in quenching of the fluorescence emission. Restoration of the fluorescence could only be achieved in the presence of complementary DNA to the original DNA probe. Subsequently, the probe–target hybridization resulted in desorption of the labeled probe into the supernatant. In their work, the authors used nanostructured ICP/PET (intrinsically conducting polymers/polyethylene terephthalate) films for the detection of *L. infantum* specific DNA sequences (Figure 2). The DNA detection system utilized PPY and PANI films immobilized with 6-carboxyfluorescein-labeled single stranded DNA (FAM-ssDNA), resulting in the quenching of the luminescence signal. Later, when the FAM-ssDNA/ICP film system was put in contact with the target ssDNA, the hybridization of the probe with its complementary target caused the formation of double-stranded DNA, release of the FAM-ssDNA from the surface of the films, and fluorescence recovery. Both types of polymeric films utilized in the study showed high sensitivity, with respective detection limits of 1.1 nM (PPY) and 1.3 nM (PANI) against the target ssDNA. Furthermore, the authors reported a linear response for the fluorescence intensity in a large concentration range of the target DNA (1–300 nM and 1–500 nM, for PPY and PANI, respectively). The FAM-ssDNA/ICP sensors showed high selectivity and could differentiate between full complementary sequences and those with just a single mismatch. They could also function in spiked complex medium, such as human blood serum, and exhibited good shelf stability. The functionalized ICP films also exhibited good shelf stability (PPY/PANI films were functional up to 180 days and probe immobilized films were functional for several days). The nanostructured ICP films described in this study show potential practical applications as strip tests for fast and sensitive fluorescent DNA detection in point-of-care clinical diagnosis protocols. This study thus describes the development of an inexpensive and simple molecular test that would not necessarily replace PCR analyses, but would rather complement them as efficient pre-screening methods. In this method, although PCR amplification is not required, both DNA extraction from a biological sample and the conversion of double-stranded DNA (dsDNA) to ssDNA are still necessary.

### 3.2. Nanomaterial as Fluorophore

Quantum dots (QDs) are nanosized semiconducting materials that have proven to be 20 times brighter and 100 times more stable compared to conventional fluorescent probes. Andreadou et al. (2016) [40] showed the use of cadmium selenite (CdSe) QDs as signal detector for the detection of *Leishmania*. Two different systems were described by this group: one for detection of *Leishmania*-specific DNA, and another for the detection of *Leishmania*-specific surface proteins (LPG and gp63). In both, the system’s magnetic beads were used to separate the analytes from the solution, whereas the presence of the targeted molecules was demonstrated by using QDs. The aim here was to develop a methodology that would be technically easy to perform, not require a specific equipment, and at the same time detect all the main pathogens of *Leishmania* spp. For the DNA-based biosensor, two highly specific DNA oligonucleotides (probe 1 and probe 2) conserved among the main leishmanial pathogens were utilized as DNA probes [68]. These DNA probes (probes 1 and 2) were biotinylated at their 5’ end and detected with streptavidin-bound QDs (15–20 nm in size). Denatured single-stranded target DNA was first put in contact with biotinylated probe 1 and was separated using the magnetic beads conjugated with streptavidin. 

**Figure 3:**
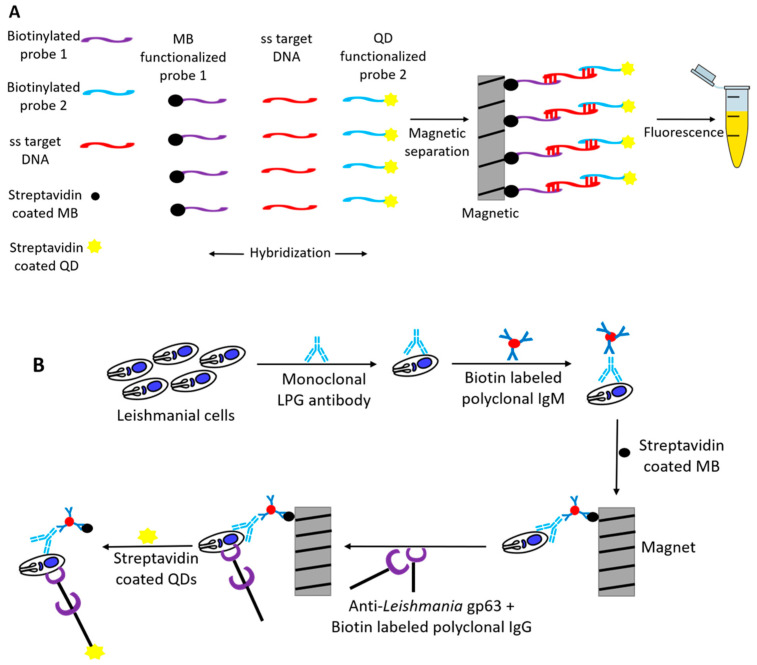
(**A**) Detection of *Leishmania*-specific target DNA using quantum dots. Target DNA was first denatured, followed by the hybridization of the functionalized magnetic beads to quantum dots via complimentary sequences. A magnetic device was then used to separate the conjugates from the solution and the positive result was detected by fluorescence. (**B**) Use of quantum dots for detecting *Leishmania*-specific proteins. Leishmanial cells were first conjugated with a monoclonal anti-*Leishmania* LPG antibody which initially bound to a biotin-labeled polyclonal anti-mouse IgM and later to streptavidin-coated magnetic beads. After separation using a magnet, the second monoclonal antibody (anti-*Leishmania* gp63) first bound to the protozoan cells and then to the biotin labeled polyclonal anti-mouse IgG. The complexes were separated again using a magnet and finally detected by streptavidin-coated quantum dots.

The separated mixture was incubated with biotinylated probe 2 and finally detected with QDs bound to streptavidin (Figure 3A). A similar methodology was used for the detection of the two different leishmanial surface antigens (LPG and gp63), using monoclonal antibodies, biotinylated IgG/IgM, and streptavidin-bound QDs. Magnetic beads conjugated with streptavidin were used as separation probe (Figure 3B). The fluorescent signal generated in both the systems was detected visually or by optical analysis (605 nm). Positive results were obtained in all the positive samples analyzed, and no fluorescent signal was obtained in the negative control samples, thus generating a sensitivity and specificity of 100%. The limit of detection was calculated to be 3.125 ng/μL and 10^3^ cells/mL for the DNA and protein methods, respectively.

### 3.3. Use of Dendrimers

Perinoto et al. (2010) [45] developed a nanostructured biosensor system to detect specific anti-*Leishmania* antibodies using capacitance measurements. In this system, phospholipid liposomes incorporating *L. amazonensis*-specific membrane antigenic proteins (proteolisosomes) together with dendrimers attached to the surface of interdigitated gold electrodes were used as the immobilized phase. The electrodes containing antigenic proteins were then used to detect antibodies. Binding of the antibodies to the electrode resulted in variations in the measured electrical response. Freshly prepared proteoliposomes were immobilized on interdigitated electrodes using the layer-by-layer technique in conjunction with polyamidoamine dendrimers 4 (PAMAM). Dendrimers, due to their branched, porous structure, are utilized for immobilizing proteins in biosensors, as they increase sensitivity and response times due to diffusion of analytes through the multilayer structure. Furthermore, the porous architecture of the film is important for confining electrical charges within its structure, which are responsible for the detectable changes in the electrical response. The PAMAM/proteoliposome electrode developed in this study could differentiate the capacitance signal between the positive and negative serum samples without showing any cross-reactivity with anti-*T. cruzi* IgGs, which are responsible for significant false positive in the current diagnostic methodologies. The capacitance-based system developed here can be extended for the diagnosis of other bacterial, protozoan, and helminth infectious diseases.

Souto et al. (2015) [46] also made use of PAMAM for detection of anti-*L. infantum* antibodies using a surface plasmon resonance (SPR)-based immunosensor. The authors used recombinant protein from *L. infantum* with unknown function (hypothetical C1 protein or C1 antigen) as a recognition element for the development of SPR immunosensor. SPR-based sensors are widely recognized as a potential analytical tool due to their extreme sensitivity to small changes in the refractive index near to the sensor surface caused by the variation of the mass on the transducer surface. SPR requires neither label nor tracer, thus reducing the number of steps for the analysis of biomolecular interactions in real time. In the current study, the immunosensor was developed by depositing self-assembled layers (SAM) of cysteamine on gold surface followed by the addition of a fourth-generation poly (amidoamine) dendrimer (PAMAM (G4)) onto which the C1 antigen was immobilized. Binding of antibody to the immobilized antigen resulted in the variation of the angle of resonance which was recorded. The authors in this work confirmed the specific binding of CVL (canine visceral leishmania) antibodies to the immobilized recombinant C1 antigen showing a limit of detection of 7.83 nmol/L. No signal was obtained for negative canine sera. A similar result was also shown by this group using different *L. infantum* antigens immobilized on SAM of 11-MUA on gold substrate for the development of a SPR immunosensor in canine serum [69].

### 3.4. Surface Plasmon Coupling

Detection of the amplification products (e.g., gel electrophoresis) after PCR amplification requires time-consuming protocols performed by trained personnel, with high cost. The aim of the study conducted by Toubanaki et al. (2016) [41] was simplification of PCR product detection, using a nucleic acid lateral flow, combined with functionalized gold nanoparticles. Gold nanoparticles, besides providing high specific surface (thus enabling the immobilization of an increased amount of bioreceptor units), were also used for visualization based on their interparticle surface plasmon coupling [70,71]. Lateral flow biosensors (LFBs) are diagnostic devices based on one time use of paper as a carrier material, where dry reagents are activated by applying a fluid sample. LFBs are important for diagnostic purposes as they are affordable, sensitive, specific, user-friendly, rapid, robust, and equipment-free [71,72]. They are an ideal platform for single use at point of care, where only a positive/negative signal is desired, with fast result time (approximately 20 min) [73,74]. In the current study [41], amplification reactions targeting kinetoplast DNA of *Leishmania* spp. Were performed on canine blood samples, and a positive signal was obtained as a red test zone on LFB. LFB consisted of an immersion pad (17 mm), a glass-fiber conjugate pad (8 mm), nitrocellulose diagnostic membrane (25 mm in length), and an absorbent pad (same as immersion pad) assembled on a plastic adhesive backing. The genomic DNA extracted from the canine sample was subjected to PCR for kinetoplast DNA amplification using biotinylated primers. Amplified product was mixed with dATP-tailed *Leishmania*-specific probe, and the mixture was applied to the conjugation pad next to the poly (dT) conjugated gold. Gold nanoparticles functionalized with oligo (dT) segments interacted with the hybridized complex of target DNA–poly (dA) probe and then were captured by immobilized streptavidin in the test zone of the LFB, indicating a positive signal (characteristic red line due to gold nanoparticle accumulation). The visual detection was completed in 20 min. Extensive optimization enabled the detection of 100 mol of target DNA. The biosensor was evaluated with actual clinical samples of infected dog blood and successfully confirmed the presence of *Leishmania*, while no product was detected for negative samples. Overall, the proposed lateral flow biosensor described by the authors is an appealing alternative platform for the detection of *Leishmania*-specific amplification products with low cost and attractive simplicity. Detection of the PCR products via LFB provides sequence confirmation by target–probe hybridization compared to size-based recognition of the amplified fragments in case of electrophoresis. The pre-hybridized sample can be applied directly on the biosensor, and only a small amount of developing solution and gold nanoparticles is required for visualization, eliminating the need of gel electrophoresis. The cost of the assay in terms of reagents and the biosensor was described to be about three euro. Furthermore, the biosensor allowed visual detection of *Leishmania*-specific amplification products within minutes without the need of any instruments. Overall, the proposed lateral flow biosensor can be considered an appealing alternative platform for *Leishmania*-specific amplification products detection with low cost and attractive simplicity. 

Sattarahmady et al. (2016) [42] reported a gold nanoparticle-based genosensor for the visual and spectrophotometric detection of non-protein-coding region of the kDNA minicircle genome from *L. major*. The mechanism was based on hybridization of AuNPs-probeDNA with a target complementary DNA sequence. Changes in the plasmonic property of AuNPs, which depends on size, surface function, and interval distance between the particles, were used to induce a color change which was used for sensing. AuNPs (16 nm) used in this study had a stable red color which turned to purple on the addition of HCl due to aggregation by acid. However, no color change was observed in the presence of complimentary sequences, probably due to the formation of double-stranded DNA, which increases the stability of the AuNP probe toward aggregation. The method described by the authors showed specificity for detection of *L. major* with a detection limit of 7.0 pg/μL. The method was evaluated with success for the detection of *L. major* genomic DNA (DNA extracts of standard cultures of *L. major*) as well as its detection in clinical samples (DNA extracted from clinical samples). Based on the same principles (AuNP and aggregation by HCl), Andreadou et al. (2014) [43] designed a genosensor using four single-stranded oligonucleotides based on conserved regions of *Leishmania* genome as probe and confirmed the specificity and sensitivity of the sensor using positive and negative control samples and the whole blood collected from dogs with suspected canine leishmaniasis. The minimum detection limit was defined to 11.5 ng/μL of target DNA sample with 100% repeatability and reproducibility.

**Figure 4:**
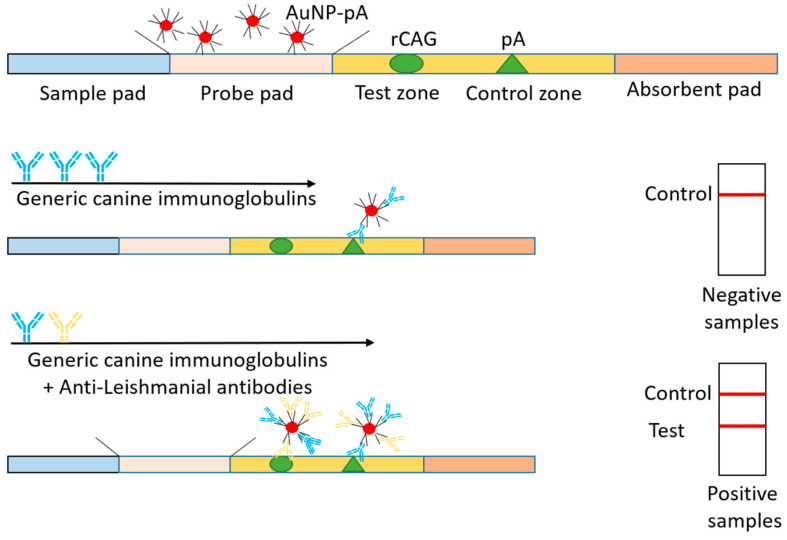
Representation of the lateral flow immunoassay (LFIA) device used for the diagnosis of visceral leishmaniasis. The strip composed of an analytical membrane contained a recombinant chimeric antigen (rCAg) and protein A (pA) which formed the test and control lines, respectively. The signal reporter included pA labelled with gold nanoparticles which are red in color due to a surface resonance band at 525 nm. AuNP-pA included in the device in dried form was pre-impregnated to the probe pad. The device also included a sample pad to adsorb the sample and distribute it uniformly to the membrane. An adsorbent pad was included to decrease the background color by increasing the volume of the sample flow. A single visible line (control) was generated for a canine serum which had no anti-leishmanial antibodies (negative sample). The presence of specific anti-leishmanial antibodies was shown by the specific binding of these antibodies to the rCAg, which produced a second red line (Test zone).

Anfossi et al. (2018) [44] also described a lateral flow immunoassay (LFIA) for quick diagnosis of canine leishmaniasis which can also be extended to other animals (Figure 4). The device used highly specific chimeric recombinant antigens (K9, K39, and K26) from the amastigote form of *L. infantum* and measured anti-leishmanial antibodies present in the serum. Protein A (from Staphylococcus known to bind to immunoglobins from various animals) labeled with gold nanoparticles (30 nm mean diameter and a SPR band at 525 nm) was used as the signal reporter, and the visual results after 15 minutes were comparable to that generated by ELISA and IFAT. The developed LFIA showed high diagnostic sensitivity (98.4%) and specificity (98.9%), in agreement with serological reference methods for diagnosing canine visceral leishmaniasis. Furthermore, the authors confirmed long thermal stability (six months of storage at room temperature or 4 °C).

### 3.5. Nanomaterials as Reducing Agents

Heli et al. (2016) [47] reported the use of a cobalt-zinc ferrite (Co_0.5_Zn_0.5_Fe_2_O_4_) magnetic QD-modified carbon electrode for the detection of a 24-mer *L. major* DNA. Methylene blue (MB) was used as an electrochemical indicator. Cobalt ferrite has been reported to interact with DNA and stabilize its structure. The cobalt-zinc ferrite quantum dots used in this study, apart from providing a high surface area for the immobilization of the ssDNA probe, also had an electrocatalytic effect toward the electroreduction of methylene blue, resulting in a higher peak current and sensitivity. Cyclic voltammograms of methylene blue were measured before and after hybridization. MB bound with both single-stranded DNA (ssDNA) and dsDNA but showed higher affinity for dsDNA. A limit of detection as low as 2 × 10 ^−19^ mol/L was observed for the complementary 24-mer target ssDNA. Similar results were also observed for *L. maior* genomic DNA with limit of detection equal to 1.8 × 10 ^−14^ ng/µL. This electrochemical genosensor was also tested with genomic DNA extracted from *L. infantum* and *L. tropica*, and DNA extracted from positive clinical samples (biopsy specimens of human samples with cutaneous leishmaniasis). The fabricated label-free, PCR-free genosensor designed in this study showed selectivity towards detection of *L. major* in both cultivated and clinical samples.

Moradi et al. (2016) [75], using a similar methodology, also designed a PCR and label-free DNA sensor using *L. maior*-specific DNA (pDNA) conjugated to gold nanoleaves, electrodeposited on gold electrodes using spermidine as shape directing agent. The methylene blue reduction peak was measured before and after hybridization of the complementary DNA. The biosensor could distinguish *L. major* from a non-complementary sequence oligonucleotide as well as the *L. tropica* genomic DNA. *L. major* in patient samples was also detected with a high selectivity. A limit of detection as low as 1.8 × 10^−20^ mol/L for the synthetic DNA and 0.07 ng/µL for the genomic DNA was recoded.

**Figure 5:**
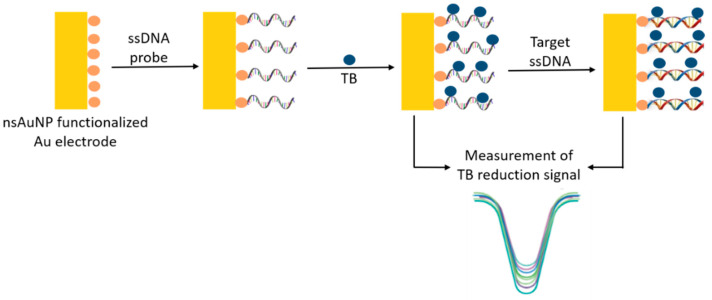
Design and functioning of an electrochemical genosensor using nsAuNP.

Similarly, Nazari-Vanini et al. (2018) [49] reported the use of an electrochemical DNA sensor for detection of the *L. infantum* genome using toluidine blue as a reduction signal (Figure 5). Non-spherical gold nanoparticles (nsAuNPs) functionalized with probe DNA (a thiolated 26-base oligonucleotide of *L. infantum* minicircle kDNA) were deposited on a gold electrode, which was tested with complementary DNA (cDNA). For this, different concentrations of cDNA were incubated with a biofunctionalized electrode for 40 min at 37 °C to conduct the hybridization. Differential pulse voltammetry (DPV) measurements were recorded before and after hybridization, and change in toluidine blue reduction signal (related to the reduction of toluidine blue bound to single stranded or double stranded DNA) was recorded. The sensor showed a linear relation with the concentration of cDNA in the range of 1 × 10^−18^ to 1 × 10^−10^ mol/L with the limit of detection of 2 × 10 ^−19^ mol/L. Higher selectivity for cDNA compared to sequences generated with 1, 2, and 3 mismatches was also recorded. The electrochemical response of the DNA sensor for extracted genomic DNA from *L. infantum* was also investigated. *L. infantum* genomic DNA showed good selectivity, and no response was observed for genomic DNA from *L. tropica* and *L. major*. In order to apply the DNA sensor for detection of the pathogen in real samples, genomic DNA from clinical samples was collected, and the resulting samples were directly analyzed by the DNA sensor without any pretreatment (e.g., PCR amplification). The developed DNA sensor in this study successfully recognized the positive samples, indicating the applicability of the DNA sensor for clinical analysis of real samples. The analytical response of the DNA sensor was observed for consecutive days to examine the stability of the sensor. The DNA sensor maintained a relatively constant response for up to 60 days with minor random fluctuations. Accordingly, the storage period of the DNA sensor was reported to be 2 months. The authors reported higher sensitivity, improved selectivity, shorter analysis time, and greater ease of fabrication and application of the developed sensor compared with the other available techniques.

Similarly, a AuNP-toluidine-based electrochemical geosensor was also recently reported by Mobed et al. (2020) [50] using *Leishmania*-specific genomic DNA. The researchers also reported very low detection limit (1ZM) and a good linear range (10^−6^ to 10^−21^ M) for cDNA detection, which is one of the striking features of this biosensor. The biosensor developed using an AuNP-modified electrode also showed selectivity (using one, two, and three mismatched nucleotides). The authors confirmed the functionality of the biosensor in human plasma samples and showed the regenerative properties of the biosensor. AuNPs played a double role in this biosensor. The terminal sulfur atoms of thiolated DNA bound to the AuNPs as a result of a stable interaction between S and Au. Furthermore, AuNPs resulted in signal amplification resulting in a good genosensor sensitivity.

**Figure 6:**
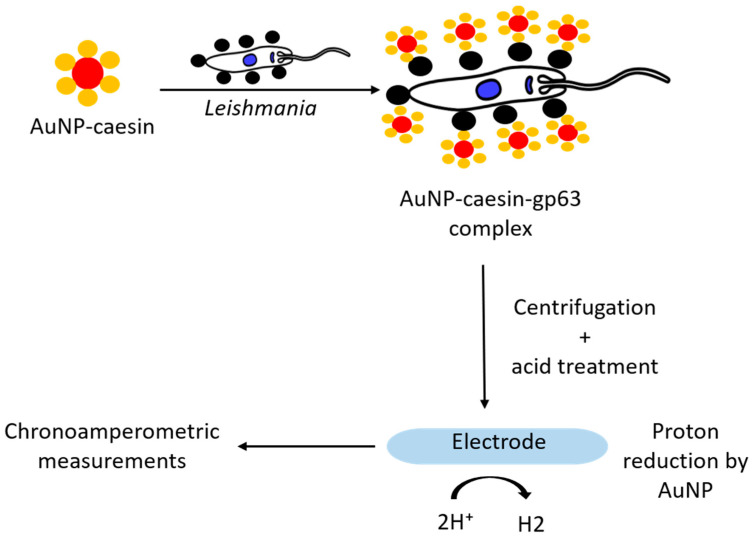
Schematic illustration of the chronoamperometric detection principle of *Leishmania infantum* parasites through hydrogen evolution reaction catalyzed by gold nanoparticles.

Surface protein gp63, expressed by both promastigote and amastigote forms of the *Leishmania*, shows the ability to degrade various substrates, such as casein, hemoglobin, gelatin, fibrinogen, and albumin [76,77]. Diouani et al. (2019) [51] described the use of AuNP-casein conjugates for the detection of *Leishmania* (Figure 6). AuNPs in this system acted both as nanocarriers and electrocatalytic labels (for catalytic reduction of hydrogen). On the other hand, conjugation of caseins with AuNPs, in addition to providing high stability to the system (ability of casein to form stable micelles), also provided these nanoparticles with biocompatible functionalities (casein-gp63 interaction). Thus, the AuNP-casein conjugate (~18 nm) was incubated with different concentrations (2 × 10^−2^ and 2 × 10^−6^ parasites/mL) of promastigotes. The formed complexes were collected by centrifugation, treated with acidic solution, and then the collected AuNPs were placed on the working electrode surface of screen-printed carbon electrodes (SPCEs) and chronoamperometric measurements were carried out. Cathodic current associated with the reduction of protons (H^+^) to hydrogen, in acidic medium, amplified by the catalytic effect of AuNPs was recorded and correlated to the concentration of AuNPs and in turn to the leishmanial parasites present in the sample. The authors describe a linear relationship between 2 × 10^−2^ and 2 × 10^5^ parasites/mL with a limit of detection of about 0.55 parasite/mL.

De la Escosura-Muñiz et al. (2016) [52] utilized isothermal amplification of kinetoplast minicircle DNA using primers labeled with AuNP and magnetic beads. The electrocatalytic activity of the AuNP (hydrogen evolution reaction) allowed rapid detection of DNA, captured using a magnet placed on the reverse side of the screen-printed carbon electrodes. PCR amplified DNA from positive sample (canine *Leishmania*) could be easily discriminated from those of healthy dogs using this methodology. Less than one parasite per milliliter of blood could be detected. This approach is thus more sensitive than traditional methods based on real-time PCR and is also more rapid, cheap, and user-friendly. 

### 3.6. Aptamers over Antibodies

Aptamers are short single-stranded RNA or DNA oligonucleotides capable of binding various biological targets with high affinity and specificity. They are isolated from randomized oligonucleotide libraries using a molecular process named SELEX (Systematic Evolution of Ligands by Exponential enrichment). Aptamers are chemically synthesized and show several advantages over antibodies such as increased stability, easy low-cost synthesis, and easy regeneration and structural modifications. Moreover, the dissociation equilibrium constants (K_d_) of aptamers are in the range of nM to pM, indicating that aptamers have a similar or greater affinity for their target than antibodies. Like antibodies, their properties are defined by the ionic conditions and pH in which they are placed. However, being shorter polymers (diameter: ~2 nm), aptamers are generally more sensitive than antibodies (diameter: ~15 nm) to their physical and chemical environment [34,78]. These properties make aptamers excellent candidates for biomedical diagnosis [71].

Despite increased advances in our understanding of the parasite biology and host–parasite interactions, protozoan parasites still affect millions of people around the world, and there is an urgent need for novel therapeutics and diagnostics options. Recent studies have generated a huge interest in aptamers as promising diagnostic tools and shown their superiority for diagnostics in comparison with other tools, essentially monoclonal antibodies that are widely used for detection of target molecules in the biomedical field [79,80,81,82]. This section will describe the aptamers selected against *Leishmania*-specific proteins which can be used instead of antibodies in diagnostic tools. One of the proposed aptasensor using the selected aptamers is also described.

A research group lead by Professor Gonzalez has used SELEX to select and characterize various aptamers targeting *L. infantum*-specific proteins such as *L. infantum*-specific histone, kinetoplast membrane protein, and Poly A Binding Protein (PAPB). All these aptamers were first isolated using respective recombinant proteins, and later their binding affinities were tested using ELONA, slot blot, and Western blot with both recombinant and endogenous proteins [83,84,85,86,87].

#### 3.6.1. *L*. *infantum* Histone-Specific Aptamers

Leishmanial parasites (together with *Trypanosoma* species) belong to the group of kinetoplastids due to the presence of a DNA-containing kinetoplast, located in the single mitochondrion at the base of the cell’s flagellum. Although histones are extremely conserved proteins, kinetoplastid core histone show sequence divergences in the amino and in the carboxy-terminal domains that can be used as potential diagnostic and/or therapeutics targets. 

**Figure 7:**
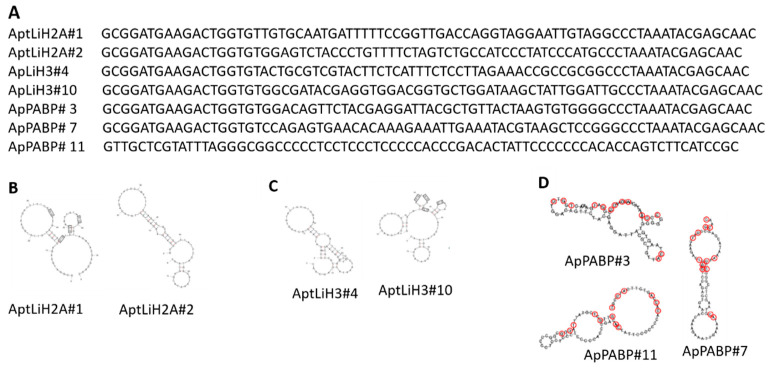
Sequence (**A**) and secondary structures of aptamers selected against *L. infantum*-specific proteins **B**: LiH2, **C**: LiH3 **D**: PAPB. The boxed G-doublets are those with the highest probability (G-Score) of participating to the G-quadruplex formation (**A**,**B**). The nucleotides labeled in red are those conserved in the sequence of the three aptamers (**C**,**D**).

So far, two aptamers (AptLiH2A#2, AptLiH2A#1) against *L. infantum*-specific H2A (LiH2A) and two aptamers (AptLiH3#4 and AptLiH3#10) against *L. infantum*-specific H3 (LiH3) have been reported by this group [84,85,87]. These aptamers were cloned and sequenced using previously selected aptamer populations (derived from ssDNA enriched population) after 3 rounds of SELEX targeting LiH2A and LiH3 recombinant proteins. Binding assays and protein interaction studies (ELONA, slot blot, and Western blot) conformed the high affinity of these selected aptamers with respective recombinant histones. At the same time, almost no binding to BSA or other leishmanial proteins was observed. Sensitivity of the aptamers to recognize LiH2A and LiH3A from *L. infantum* promastigotes was also confirmed in the cell lysates. Binding was confirmed in case of total proteins and nuclear fractions with no reactivity against cytoplasmic fractions. Their binding efficiencies and limit of detection are shown in Table 2. Structural analysis of the aptamer sequences using the program mFold and QGRS-mapper showed typical stem and loop motifs with higher probability of a G-quadruplex structure for the aptamers AptLiH2A#1Lih2A and AptLiH3#10. Their sequences, secondary structures, and lowest free energies are shown in Figure 7. In the two aptamers (AptLiH2A#1 and AptLiH2A#2) targeting LiH2A, high affinity binding was shown only with peptides 5 and 8 of LiH2A protein. (*p* < 0.01; ANOVA followed by Dunnett’s test). Similar studies with LiH3 showed that aptamers AptLiH3#4 and AptLiH3#10 recognize the region between the amino acids 21 to 60, corresponding to peptides 3, 4, and 5, with high affinity (*p* < 0.001; ANOVA followed by Dunnett’s test), which correspond to the amino terminal domain with the highest sequence variability and presence of antigenic determinants of the protein.

#### 3.6.2. Aptamer Targeting Poly-A Binding Protein 

The Poly-A Binding Protein (PABP) is a conserved eukaryotic polypeptide involved in many cellular functions linked with the metabolism of messenger RNAs. Different PABPs have been described in *T. cruzi*, *T. brucei*, *L. major*, and *L. amazonensis*. The PABP homolog from *L. infantum*, called LiPABP, shows conserved domains present in other PABPs and maintains poly (A) binding properties. Three aptamers—ApPABP#3, ApPABP#7, and ApPABP#11—were selected after four rounds of SELEX against rLiPAPB protein [86]. The aptamers were able to recognize the recombinant protein with affinity in the low nanomolar range (5–10 nM). No binding to BSA was observed during similar conditions. Slot blot assays showed that only 50 nM of each of the three aptamers could detect as low as 6.25 ng of LiPABP. The secondary structure of the aptamers was analyzed by using the Rapidshape program as shown in Figure 7. Based on their free energy value, ApPABP#7 appears to be the more stable aptamer (ΔG = −14.00 kcal/mol), followed by ApPABP#11 (ΔG = −9.30 kcal/mol) and ApPABP#3 (ΔG = −7.40 kcal/mol.). 

#### 3.6.3. Aptamer against Kinetoplast Surface Protein

**Figure 8:**
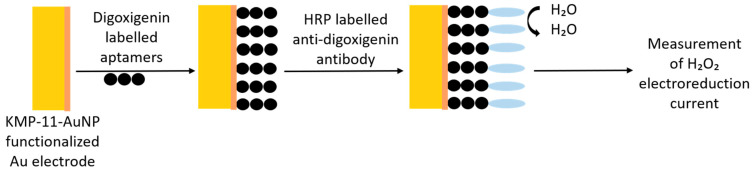
Scheme of the proposed KMP-11 aptasensor.

Kinetoplastid membrane protein-11 (KMP-11) is a major component of the cell membrane of kinetoplastid parasites. Moreno et al. (2003) [83] described a pool of ssDNA aptamers against *L. infantum* gold-labeled recombinant KMP-11 (LiKPM-11). Using dot blot ELISA and Western blot assays, the authors showed that the aptamer population obtained after ten rounds of SELEX showed selective binding to the rKMP-11 and total proteins from *L. infantum*. No binding to BSA and total proteins from *Escherichia coli* were observed. The same authors further checked the functionality of this interaction using a electrochemical biosensor (Figure 8) [53]. LiKMP-11-functionalized gold nanoparticles were electrodeposited on arrayed gold microelectrode. The previously selected aptamer population was digoxigenin-labeled, and electrochemical measurements were recorded using an anti-digoxigenin antibody labeled with horseradish peroxidase. BSA-gold nanoparticles electrodes were used as negative control. The response of the biosensor to different LiKMP-11 concentration was measured using a displacement assay, where the electroporated microelectrodes were first incubated with a digoxigenin-labeled aptamer population and later put in contact with different concentrations of LiKMP-11 (0, 0.05, 0.25, and 0.5 mg/mL) for 1 h. After two washes with SELEX buffer, the electrodes were incubated with anti-digoxigenin antibody labeled with horseradish peroxidase in SELEX buffer, and the H_2_O_2_ electroreduction currents were measured. The authors obtained a linear relationship between free LiKMP-11 concentration and the intensity of the electrochemical reduction current. A limit of detection of 25 mg/mL (2.27 mM) of LiKMP-11was achieved, which, although being very high, demonstrates the functionality of the current approach towards the development of an aptasensor for the diagnosis of leishmanial parasites.

## 4. Conclusions

Rapid and accurate diagnosis is of extreme importance for the control of leishmaniasis. Different species of *Leishmania* present variable aspects of virulence and pathogenicity. This, together with the host genetics, result in the wide clinical diversity of leishmaniasis, which makes the diagnosis of the disease more complex. Moreover, the clinical symptoms observed in VL are similar to that of different diseases common in areas where leishmaniasis is endemic, so that a specific diagnostic method is required to avoid cross-reactivity with other diseases. The most specific methods for the diagnosis of *Leishmania* spp. are currently available in reference hospitals or research centers, so that the most used field assays detect only a few species of *Leishmania*, not being suitable for regions with several endemic species. Therefore, there is a need to develop more accurate and accessible tests, as well as sensitive tests for patients co-infected with HIV and for asymptomatic infections, besides effective biomarkers for monitoring patients who have undergone different treatment regimens or who have had recurrences. Many new techniques are being studied, with the aim to promote improved diagnosis. Several cost-effective, convenient biosensors have been described over the years for the rapid and accurate diagnosis of Leishmania. These also show high sensitivity and longer durability. A number of engineered biosensors making use of excellent conduction, catalytic, optical, and structural properties of nanomaterials are already in used for diagnosis and imaging in health sector for diagnosis of diseases like cancer. Nevertheless, there are still doubts and opportunities for improving these methods. More research aiming to improve the efficiency of these methods and the standardization of steps are necessary. Besides, costs must be evaluated carefully as leishmaniasis is basically a disease of poor countries. Published research describes the use of many inorganic materials like carbon nanotubes, graphene oxide, gold, silver, quantum dots, and conductive polymers for the design of diagnostic tools. These materials can link specific antigens, thus improving probe–target interaction. The use of the nanomaterial for signal amplification has also been well explored. However, further improvements are required with respect to the specificity of the transduced signal evicting cross-reactions. The use of conducting, catalytic, optical, and structural properties of nanomaterials are promising; however, the question is: Is this technique is viable to be used at large scale with an effective cost–benefit ratio? Most of the recognition elements in biosensors described in this review showed the use of antibodies with only one study making use of aptamers. Aptamers are highly promising with superior properties compared to antibodies. Selection of new, more sensitive aptamers with better and more systematic characterization is definitely desired. Moreover, most of the aptamer-based biosensors rely on simple binding and are prone to nonspecific binding, especially in a complex sample matrix. An electrochemical DNA (E-DNA) sensor platform could be a good alternative, where signaling does not rely on simple absorption of the target on the sensor, thus the system performs well even in a complex medium. Sensors based on aptamer conformational change or catalysis could thus be more accurate.

## Figures and Tables

**Table 1:** Nano-based biosensors described for the detection of *Leishmania*.

Type of Biosensor	Nanomaterial Used	Type of Pathogen	Target Biomolecule	Limit of Detection	References
Genosensor/Optical	PPY PANI	*L. infantum*	FAM-ssDNA	1.1 nM ^†^ 1.3 nM	[23]
Genosensor/Optical	cadmium selenite QD	*Leishmania* spp.	Conserved specific genomic DNA LPG and gp36 antigens	3.125 ng/μL ^‡^	[40]
	
	
	
Immunosensor/Optical				10^3^ cells/mL ^¥^	
Genosensor/Optical	AuNP	*L. infantum*	KinetoplastDNA	100 fmol	[41]
Genosensor/Optical	AuNP	*L. major*	KinetoplastDNA	7.0 pg/μL	[42]
Genosensor/Optical	AuNP	*Leishmania* spp.	KinetoplastDNA	11.5 ng/μL	[43]
Immunosensor/Optical	AuNP	*L. infantum*	Chimeric recombinant antigens (K9, K39, and K26)	-	[44]
Immunosensor/Impedimetric	PAMAM	*L. amazonensis*	membrane proteins	10^−5^ mg/mL	[45]
Immunosensor/SPR	PAMAM	*L. infantum*	hypothetical C1protein	7.83 nmol/L	[46]
Genosensor/Electrochemical	cobalt-zinc ferrite QD	*L. major*	KinetoplastDNA	1.8 × 10^−14^ ng/µL	[47]
Genosensor/Electrochemical	AuNP	*L. major*	*L. major* specific DNA	1.8 × 10^−20^ mol/L 0.7 ng/µL	[48]
Genosensor/Electrochemical	AuNP	*L. infantum*	KinetoplastDNA	2 × 10^−19^ mol/L	[49]
Genosensor/Electrochemical	AuNP	*Leishmania* spp.	Leishmania specific DNA	1ZM	[50]
Casein-gp63 interaction/Electrochemical	AuNP	*L. infantum*	gp63 surface protein	0.55 parasite/mL	[51]
Genosensor/Electrochemical	AuNP	*Leishmania* spp.	KinetoplastDNA	0.8 parasite/mL	[52]
Aptasensor/Electrochemical	AuNP	*L. infantum*	Kinetoplastid membrane protein-11	2.27 mM	[53]

PPY—polypyrrole, PAN—polyaniline, QD—quantum dot, PAMAM—polyamidoamine dendrimer, AuNP—gold nanoparticle, FAM-ssDNA—6-carboxyfluorescein-labeled single stranded DNA; ^†^, In this case, the value represents the concentration of DNA as the target molecule is ssDNA from *L. infantum*; ^‡^ In this case, the value represents the concentration of DNA as target molecule is genomic DNA; ^¥^, In this case, the value represents the number of parasites.

**Table 2:** Aptamers selected against *Leishmania*-specific proteins.

Aptamer	Target Protein	Organism	Affinity (K_d_)	Minimum Number of Detectable Promastigotes	References
AptLiH2A#2, AptLiH2A#1	H2A	*L. infantum*	0.96 ± 0.17 nM	7500	[85]	
			1.16 ± 0.28 nM	7500	
AptLiH3#4 AptLiH3#10	H3	*L. infantum*	0.52 ± 0.05 nM	6000	[87]
			0.37 ± 0.05 nM	9000	
ApPABP#3	PAPB	*L. infantum*	5.4 ± 1.1 nM	2500	[86]
ApPABP#7			6.0 ± 2.6 nM		
ApPABP#11			10.8 ± 2.7 nM		
Aptamer population	KMP-11	*L. infantum*	-	-	[83]

## Data Availability

Not applicable.
